# Naa10p impairs PGC‐1α/Pparγ2 interaction to inhibit mitochondrial protection in pancreatitis

**DOI:** 10.1002/ccs3.70015

**Published:** 2025-06-23

**Authors:** Jie Du, Hai Jiang, Taizhe Zhang, Chuanming Zheng, Zhong Ji

**Affiliations:** ^1^ Department of Emergency Surgery The First Affiliated Hospital of Bengbu Medical University Bengbu China

**Keywords:** acute pancreatitis, N‐terminal acetyltransferase 10, peroxisome proliferator‐activated receptor gamma coactivator 1‐alpha, Pparγ2, uncoupling protein 1

## Abstract

Naa10p disrupts the protective mitochondrial UCP1 pathway in acute pancreatitis (AP). This study demonstrates that Naa10p upregulation in AP correlates with decreased UCP1 expression and increased reactive oxygen species production. Silencing Naa10p improved cell survival, suppressed inflammation, and enhanced UCP1 levels by promoting PGC‐1α/Pparγ2 interaction. Co‐immunoprecipitation and luciferase assays confirmed that Naa10p inhibits UCP1 promoter activation. This study reveals the significance of Naa10p as a potential target for the treatment of AP and provides a new idea for the intervention of pancreatic inflammatory diseases.

## Graphical abstract

This study identifies Naa10p as a key regulator in acute pancreatitis, linking its elevated expression to decreased UCP1 levels, mitochondrial dysfunction, and enhanced inflammation. Silencing Naa10p mitigates these effects, enhancing cell survival and reducing oxidative stress. These findings highlight Naa10p as a potential therapeutic target for pancreatitis.

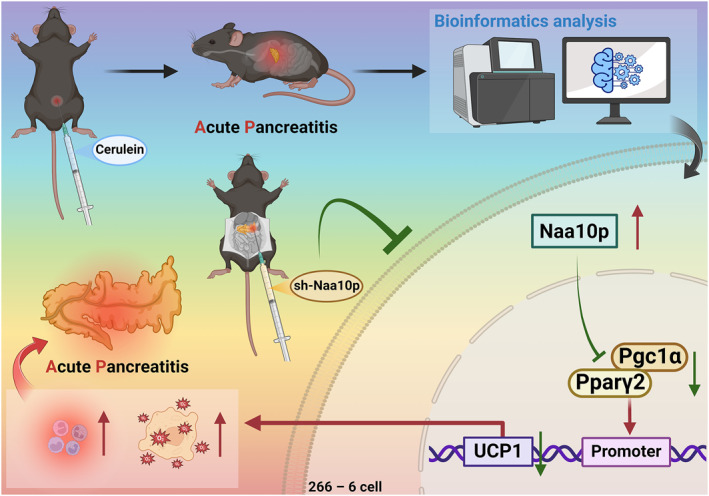

## 1 INTRODUCTION

Acute pancreatitis (AP) is a severe inflammatory disease characterized by localized tissue damage to the pancreas and systemic inflammatory responses,^1^, ^2^ which can lead to multiple organ failure and even death in severe cases. Pancreatitis includes both acute and chronic forms and is recognized as a complex inflammatory disease.^3^ AP typically presents abruptly with upper abdominal pain, nausea, vomiting, and bloating, whereas chronic pancreatitis is associated with recurrent abdominal pain and progressive pancreatic dysfunction.^3^ The pathogenesis of AP is complex,^4^ involving autodigestion, oxidative stress, and apoptosis, yet the detailed molecular mechanisms are not fully understood.^5^ Current treatments include supportive care, endoscopic interventions, and pharmacological therapy, aiming to relieve symptoms, prevent complications, and improve quality of life.^6^ However, these treatments are insufficient for curing chronic pancreatitis and remain limited in addressing complications of AP. With the rapid advancements in molecular biology and genomics, researchers have been employing high‐throughput technologies to explore the pathological processes of pancreatitis,^7^ aiming to identify new therapeutic targets.^8^ The treatment of pancreatitis presents significant challenges^9^ due to the complexity of its pathogenic mechanisms and the urgent need for timely intervention, necessitating in‐depth studies of its molecular mechanisms to develop effective intervention strategies.^4^ Therefore, it is of significant importance to clarify the pathogenesis of AP and identify new therapeutic targets to improve clinical outcomes.

In the regulation of cellular metabolism and energy balance, PGC‐1α (peroxisome proliferator‐activated receptor gamma coactivator 1‐alpha) and UCP1 (uncoupling protein 1) are two key proteins.^10^, ^11^ PGC‐1α is a transcriptional coactivator widely involved in regulating energy metabolism, particularly in mitochondrial biogenesis and oxidative phosphorylation.^12^ UCP1, primarily expressed in brown adipose tissue, regulates thermogenesis and overall energy balance by promoting energy expenditure.^13^ Recent studies suggest that these proteins may also regulate inflammatory responses, mainly by controlling inflammatory cell activity by modulating metabolic and oxidative stress balance.^14^

N‐terminal acetyltransferase 10 (Naa10p) is an enzyme ubiquitously present in mammalian cells, responsible for catalyzing the acetylation of protein N‐termini, which influences protein stability, localization, and function. Naa10p is crucial in various biological processes, including cell cycle regulation, signal transduction, and cellular responses to external stimuli.^15^, ^16^ Recent studies have also identified the key role of Naa10p in modulating cellular responses to inflammatory stimuli by affecting the expression of inflammation‐related genes.^17^ However, the specific role and mechanisms of Naa10p in AP remain unclear, making further research in this area critical for understanding new therapeutic approaches for pancreatitis. Previous studies have demonstrated that Naa10p acetylates the N‐terminus of PGC‐1α, thereby preventing its interaction with Pparγ2 and suppressing the expression of UCP1, a key gene involved in beige adipocyte function.^18^

To explore the function of Naa10p in AP, this study employed various modern biotechnological approaches. We first established an AP model in C57BL/6J mice through intraperitoneal injection of cerulein to induce pancreatitis.^19^, ^20^ Subsequently, we analyzed gene expression differences in pancreatic tissues before and after treatment using RNA sequencing (RNA‐seq). Key genes and signaling pathways were identified using weighted gene coexpression network analysis (WGCNA) and machine learning methods. Further, the interactions among Naa10p, PGC‐1α, and Pparγ2 in regulating UCP1 gene expression were confirmed through ChIP‐qPCR, luciferase reporter assays, and co‐immunoprecipitation (Co‐IP) experiments.

The objective of this study was to reveal how Naa10p modulates inflammatory responses in AP by influencing the PGC‐1α/UCP1 signaling axis. The findings indicate that Naa10p is upregulated during the progression of pancreatitis. Silencing Naa10p significantly enhanced UCP1 expression, alleviated inflammation and oxidative stress, and promoted pancreatic cell proliferation while inhibiting apoptosis and necrosis. These results highlight the potential therapeutic role of Naa10p in pancreatitis and provide a new molecular target for the treatment of inflammatory diseases, with significant scientific and clinical implications.

## 2 MATERIALS AND METHODS

### 2.1 Plasmid construction and selection

Using RNA interference technology, specific shRNA target sequences for the Naa10p gene were designed based on its mRNA sequence available in the NCBI database. Expression vectors LV‐sh‐Naa10p‐1, LV‐sh‐Naa10p‐2, LV‐sh‐Naa10p‐3, and the control vector LV‐sh‐NC were constructed. The vector demonstrating the highest silencing efficiency was selected for subsequent cellular experiments. Additionally, a fragment of Naa10p was inserted into the LV‐Naa10p‐V5 vector to create an overexpression vector, LV‐Naa10p, along with a corresponding control vector, lentiviral negative control vector (LV‐NC). All lentiviral vectors were purchased from GenePharma. The sequences used for silencing are provided in Supporting Information S1: Table S1.

### 2.2 Experimental animals and grouping

Healthy four‐week‐old C57BL/6J mice, weighing 20–25 g and of specific‐pathogen‐free status, were purchased from Chengdu Dossy Experimental Animals Co. Ltd. The mice were divided into a normal control group (Con group) and an AP model group (AP group). All mice underwent a 2‐week acclimation period before the onset of experiments.

For the in situ injection procedure, mice were anesthetized with 1% sodium pentobarbital (50 mg/kg). A 2‐cm incision was made on the upper left side of the abdomen to expose the tail of the pancreas and spleen. Subsequently, at least 5 × 10^9^ IFU/mL of lentivirus (100 μL, totaling at least 5 × 10^8^ IFU) was injected into multiple sites in the tail of the pancreas. Mice were allowed to recover at 37°C until fully awake. Seven days postsurgery, the AP model was induced by intraperitoneal injection of cerulein.^21^ Mice in the AP model group received intraperitoneal injections of cerulein (100 μg/kg, HY‐A0190, MCE) every hour for 8 injections. In contrast, the normal control group received an equivalent volume of phosphate‐buffered saline (PBS).^22^ To dynamically assess organ injury after model induction, we measured serum biochemical markers—total protein, lipase, alanine aminotransferase (ALT), and lactate dehydrogenase (LDH)—at 6, 12, and 24 h postinjection. In addition, we collected 24‐h urine samples using metabolic cages to measure urinary creatinine, and we analyzed serum creatinine levels 24 h postinjection to evaluate acute kidney injury.

The experimental groups were as follows: Con group (normal control, *n* = 16), AP group (AP model mice, *n* = 16), AP+sh‐NC group (AP model mice with the tail of the pancreas injected with sh‐NC lentivirus, *n* = 6), and AP+sh‐Naa10p group (AP model mice with the tail of the pancreas injected with sh‐Naa10p lentivirus, *n* = 6). Pancreatic tissues and serum samples were collected for analysis. All animal experiments complied with the animal ethics committee's approval and followed animal protection, welfare, and ethics principles.

### 2.3 α‐Amylase content determination

Serum or cell culture supernatants were collected, and α‐amylase levels were measured using a mouse α‐amylase ELISA kit (BG‐MUS10009, Novatein Biosciences) according to the manufacturer's instructions.

### 2.4 Histological analysis

Pancreatic tissues were collected from mice and immediately fixed in 10% neutral buffered formalin for 24 h. After fixation, the tissues were dehydrated, cleared, and embedded in paraffin. Continuous sections of 4 μm thickness were prepared for subsequent histological analysis. Paraffin sections were deparaffinized with xylene and rehydrated through a graded ethanol series. Sections were stained with hematoxylin for 5 min, rinsed in tap water, differentiated briefly in 1% hydrochloric acid ethanol, and then blued. Eosin staining was applied for 1–3 min, followed by a rinse in running water. After staining, the sections were dehydrated through graded ethanol, cleared in xylene, and mounted with neutral balsam.

Severity scoring of pancreatitis: Two independent investigators, blinded to the experimental conditions, assessed the severity of pancreatitis by examining hematoxylin and eosin (H&E)‐stained sections under a microscope. Severity was scored based on a standardized scoring system, including parameters such as inflammatory cell infiltration, acinar cell necrosis, hemorrhage, and edema. An average score was calculated for each sample to ensure objectivity and consistency.

Masson staining analysis: After H&E staining, pancreatic tissue sections were further subjected to Masson staining to assess fibrosis in pancreatitis. This method highlights collagen deposition, staining it blue or green, whereas cells and muscle appear red or pink. After staining, sections were dehydrated through graded ethanol, cleared in xylene, and mounted with neutral balsam. Microscopic examination was used to quantify collagen distribution and assess fibrosis severity.

### 2.5 Detection of reactive oxygen species levels in pancreatic tissues and cells

According to the manufacturer's instructions, reactive oxygen species (ROS) levels in pancreatic tissues were assessed using dihydroethidium staining (S0063, Beyotime). ROS levels in cells were evaluated using a ROS detection kit (S0033S, Beyotime) following the provided protocol. Three random fields were selected for each sample, and images were captured with a fluorescence microscope. The fluorescence intensity of the stained images was quantified using the ImageJ software.

### 2.6 RNA extraction and sequencing

Pancreatic tissues were collected from control and pancreatitis mice, with 10 samples per group. Total RNA was extracted using Trizol reagent (15596026, Invitrogen). RNA concentration and purity were determined using a NanoDrop 2000 spectrophotometer (1011U, NanoDrop). Only RNA samples meeting the following criteria were used for further analysis: RNA integrity number ≥ 7.0 and a 28S:18S ratio ≥ 1.5.

Sequencing libraries were prepared and sequenced by CapitalBio Technology, with 5 μg of RNA used per sample. Briefly, ribosomal RNA was removed from total RNA using the Ribo‐Zero magnetic kit (MRZE706, Epicentre Technologies). Libraries for sequencing were constructed using the NEBNext Ultra RNA Library Prep Kit (#E7775, NEB) for Illumina. RNA was fragmented in NEBNext First Strand Synthesis Reaction Buffer (5×) to approximately 300 base pairs (bp). First‐strand cDNA synthesis was performed using reverse transcriptase and random primers, followed by second‐strand cDNA synthesis in the dUTP Mix (10×) reaction buffer for strand specificity. cDNA fragments were end‐repaired, poly(A)‐tailed, and ligated with sequencing adapters. Illumina sequencing adapters were then added, and the second strand of cDNA was digested using USER Enzyme (#M5508, NEB) to construct a strand‐specific library. The library DNA was amplified, purified, and enriched by polymerase chain reaction (PCR). Finally, the library was validated with an Agilent 2100 and quantified using the KAPA Library Quantification Kit (KK4844, Kapa Biosystems). Paired‐end sequencing was conducted on the NextSeq CN500 platform (Illumina).

### 2.7 Quality control of sequencing data and alignment to reference genome

The quality of paired‐end reads from the raw sequencing data was assessed using FastQC software v0.11.8. The raw data were processed using Cutadapt v1.18 to remove Illumina sequencing adapters and poly(A) tails. A custom Perl script eliminated reads with an N content greater than 5%. The FASTX Toolkit v0.0.13 was then used to retain reads with a base quality score greater than 20 for at least 70% of bases. BBMap software was employed to repair paired‐end sequences. Finally, the filtered high‐quality reads were aligned to the mouse reference genome using Hisat2 v0.7.12.

### 2.8 Differential expression analysis

The obtained mRNA expression data were normalized, with log_2_ transformation and quantile correction performed using the limma package (v3.48.3) in R to minimize batch effects and technical noise. Differential expression analysis was conducted using DESeq2 (v1.32.0) on the normalized data to identify significantly upregulated or downregulated mRNAs in pancreatitis mice. The criteria for differential expression were set at a *p*‐value < 0.05 and |log_2_FoldChange| > 1.

### 2.9 WGCNA

We calculated the median absolute deviation (MAD) for each gene in the expression profile and excluded 50% of genes with the lowest MAD values. Outlier genes and samples were removed using the goodSamplesGenes method in the “WGCNA” R package. Next, we constructed a scale‐free coexpression network with WGCNA, setting the minimum size of gene dendrograms to 100 and the sensitivity parameter to 15. Modules with a distance less than 0.5 were merged, resulting in four coexpression modules. Pearson correlation analysis was then used to examine the association between each module and the experimental groups. The module most significantly correlated with osteosarcoma was selected as the disease‐related gene module for subsequent analysis.

### 2.10 Least absolute shrinkage and selection operator regression algorithm

In our bioinformatics study, we applied the least absolute shrinkage and selection operator (LASSO) regression to identify key genes associated with the disease. We set a random seed to ensure experimental reproducibility and used the glmnet package to process the dataset with many variables. Candidate disease‐related genes were modeled using the glmnet function for binary classification, with sample categories extracted as response variables through regular expression. Model evaluation was conducted by plotting the model object and using cv.glmnet for cross‐validation to determine the optimal lambda value. The genes corresponding to nonzero coefficients at the optimal lambda value were identified as critical genes associated with the disease state and were selected for further analysis.

### 2.11 Gene ontology and Kyoto Encyclopedia of Genes and Genomes enrichment analysis

Gene ontology (GO) and Kyoto Encyclopedia of Genes and Genomes (KEGG) enrichment analyses were conducted on the intersecting genes using the “clusterProfiler” package in R, accessed through the Xiantao Academic online platform. A significance threshold of *p* < 0.05 was set to identify significantly enriched cellular functions, signaling pathways, and disease‐associated pathways of the intersecting genes. Based on *p*‐values, the KEGG enrichment analysis was performed with “clusterProfiler” in R, and the results were visualized with a bubble plot to illustrate the KEGG enrichment pathways.

### 2.12 Cell culture and grouping

266‐6 cells (YC‐C014, Ubigene) were cultured in high‐glucose Dulbecco's Modified Eagle Medium supplemented with 10% fetal bovine serum at 37°C in a 5% CO_2_ incubator. For the in vitro pancreatitis model (AP group), cells were treated with 1 μmol/L cerulein for 24 h, whereas control cells were treated with an equal volume of PBS.^23^ 266‐6 cells are widely used in AP research due to their retention of key acinar characteristics, including responsiveness to secretagogues (e.g., carbachol) that triggered premature activation of digestive enzymes, leading to cellular damage, inflammation mediator release, and cell death. Their stable growth and gene editing adaptability make them ideal for studying molecular mechanisms and screening therapeutic agents in AP.^24^, ^25^

The in vitro AP model of 266‐6 cells was divided into four groups: the AP+sh‐NC group (AP model transfected with sh‐NC lentivirus), the AP+sh‐Naa10p group (AP model transfected with sh‐Naa10p lentivirus), the AP+oe‐NC group (AP model transfected with Lv‐NC lentivirus), and the AP+oe‐Naa10p group (AP model transfected with Lv‐Naa10p lentivirus). Cells were seeded at a density of 3 × 10^5^ cells per well in six‐well plates. When cell confluency reached 80%, Opti‐MEM (Gibco) medium was added with 5 × 10^9^ IFU/mL of lentivirus (100 μL, with a total viral load of at least 5 × 10^8^ IFU) and gently mixed before adding to each well. Cells were incubated in a 37°C, 5% CO_2_ incubator for 48 h. After infection, the medium was replaced with a regular culture medium to remove the residual virus, and the cells were selected with appropriate antibiotics to establish stably infected cell lines. Selected cell lines were treated with 10 nmol/L cerulein for 24 h for subsequent experimental observations.^26^

The PGC‐1α wild type Flag and PGC‐1α A1P Flag plasmids were transfected into 266‐6 cells using Lipofectamine 2000 transfection reagent (Invitrogen). Before transfection, 266‐6 cells were seeded at a density of 5 × 10^5^ cells per well in six‐well plates. When cell confluency reached 80%–90%, transfection was initiated. Following the manufacturer's instructions, 1 μg of plasmid DNA and 3 μL of Lipofectamine 2000 were diluted separately in Opti‐MEM medium, mixed, and incubated at room temperature for 20 min to form DNA‐reagent complexes. These complexes were then added to each well, gently mixed, and incubated at 37°C in a 5% CO_2_ incubator for 48 h. After 48 h, the medium was replaced with a fresh culture medium for subsequent experiments. Plasmids were purchased from GenScript.^18^

### 2.13 EdU assay

Cells were digested with trypsin, gently dispersed into single cells, and counted. Five thousand cells per well were seeded into a 96‐well plate with 100 μL of culture medium per well. When cells reached approximately 70% confluency, transfection and corresponding treatments were performed in five replicates per group. At 24, 48, 72, and 96 h post‐treatment, 100 μL of 50 μM 5‐Ethynyl‐2′‐deoxyuridine (EdU) solution (RiboBio, Cat# C10310) was added per well. Wells with only EdU and culture medium served as blank controls. After 2–4 h of incubation, cells were stained with Apollo fluorescent dye and analyzed.

### 2.14 Flow cytometry analysis

After transfection, cells were cultured for 24 and 48 h. The culture medium was discarded, and the cells were washed once with PBS. Cells were then digested with 0.25% trypsin solution, and digestion was stopped by adding a serum‐containing medium once the cells appeared rounded and detached under the microscope. Cells were gently pipetted to form a single‐cell suspension and centrifuged at 1000 r/min for 5 min, with the supernatant discarded. Cells were washed twice with PBS and filtered through a 60 μm mesh. The cells were fixed with precooled 70% ethanol for 30 min, centrifuged, and washed with PBS. An apoptosis and necrosis detection kit (C1056, Beyotime) was used for staining. After staining, the sample volume was adjusted to 1 mL and filtered again through a 60 μm mesh.

Samples were then analyzed on a BD Aria flow cytometer (FACS Calibur, Beckman Coulter) to assess the cell cycle, with three samples per group, each in triplicate. In dual‐staining analysis, live cells exhibited weak red and blue fluorescence, early apoptotic cells displayed weak red and robust blue fluorescence, and late apoptotic and necrotic cells showed solid red and blue fluorescence (Annexin V+/PI+ cells).

### 2.15 ChIP‐qPCR

266‐6 cells from each group were fixed with 1% formaldehyde for 10 min and quenched with 0.125 M glycine for 5 min. Cells were scraped and lysed in 1% sodium dodecyl sulfate, 10 mM EDTA, and 50 mM Tris‐HCl (pH 8.0). DNA was sonicated to fragments of approximately 150–300 bp (Bioruptor, Diagenode). Immunoprecipitation was conducted overnight at 4°C with 3 μg of either rabbit polyclonal anti‐PGC‐1α antibody (sc‐518038, Santa Cruz) or anti‐Pparγ1 antibody (sc‐81152, Santa Cruz). Protein G agarose beads were used to capture antibody‐bound chromatin, followed by washing, elution, and reverse cross‐linking. ChIP DNA was then extracted using phenol/chloroform and precipitated with ethanol. Quantitative PCR analysis of immunoprecipitated DNA was performed on a LightCycler 480 II (Roche). Primers targeting the promoter region of the UCP1 gene were as follows: F: 5′‐GGGTGCTAGGGACTAAAGGTG‐3′ and R: 5′‐TCTACTGATACCTGGCTCAGC‐3′.

### 2.16 Luciferase reporter assay

For the luciferase reporter assay, 266‐6 cells at 60% confluence were transfected in six‐well plates using Lipofectamine 2000 (Thermo Fisher Scientific) with plasmids expressing PGC‐1α, Pparg2, or sh‐Naa10p. All plasmids were purchased from GenePharma. Cells were harvested 72 h post‐transfection. Each 5 μL cell lysate was mixed with 100 μL of substrate buffer (Firefly and Renilla Luciferase Assay Kit; RG005, Beyotime) to measure luciferase activity.

### 2.17 Co‐IP assay

Proteins were isolated from each group of 266‐6 cells. A total of 500 μg of protein was incubated overnight with 4 μg of anti‐PGC‐1α antibody (sc‐518038, Santa Cruz) in lysis buffer containing 50 mM Tris‐HCl, 150 mM NaCl, 1% Triton X‐100, and a protease and phosphatase inhibitor cocktail (PPC1010, Sigma). The antibody was preconjugated to either dimethyl pimelimidate dihydrochloride or 25 μL of anti‐Flag M2 magnetic beads (M8823, Sigma) and protein A/G magnetic beads, followed by washing in lysis buffer. Immunoprecipitated PGC‐1α was eluted with 0.1 M glycine‐HCl (pH 2.5) and analyzed by western blot to assess Co‐IP with Pparγ1/2 and Naa10p.

### 2.18 Tetracycline‐induced system for evaluating PGC‐1α‐A1P regulation of UCP1 expression

To investigate the role of PGC‐1α and its mutant A1P in regulating the thermogenesis‐associated gene UCP1, 3xFlag‐tagged plasmids encoding PGC‐1α and PGC‐1α.A1P (pAS4.1w.Pbsd‐aOn‐PGC‐1α‐3xFlag and pAS4.1w.Pbsd‐aOn‐PGC‐1α.A1P‐3xFlag) were constructed and expressed via a doxycycline (Dox)‐inducible system. RT‐qPCR quantified UCP1 mRNA levels to verify the transcriptional activation by PGC‐1α and PGC‐1α.A1P. Additionally, western blot was used to analyze the expression levels of PGC‐1α‐Flag, UCP1, Naa10p, and GAPDH proteins, assessing the impact of the PGC‐1α mutant on thermogenesis‐related gene expression.

### 2.19 Western blot analysis

Total protein was extracted from adipose tissue and cells using protein lysis buffer (Catalog No. C0481, Sigma), and protein concentrations were quantified using the Bicinchoninic Acid assay method. Proteins were separated on 10% sodium dodecyl sulfate polyacrylamide gel electrophoresis gels with 20 μg of protein loaded per lane. Samples were mixed with loading buffer, boiled at 100°C for 5 min, cooled on ice, centrifuged, and loaded into lanes for electrophoresis. The separated proteins were then transferred to a nitrocellulose membrane.

Membranes were blocked with 5% skim milk for 1 h, followed by overnight incubation at 4°C with primary antibodies: Naa10p (ab194297, Abcam, 1:2000), UCP1 (ab23841, Abcam, 1 μg/mL), PGC‐1α (ab313559, Abcam, 1:1000), Pparγ1/2 (ab310323, Abcam, 1:1000), IL‐6 (ab290735, Abcam, 1:1000), TNF‐α (ab183218, Abcam, 1:1000), Flag (ab205606, Abcam, 1:1000), and GAPDH (ab8245, Abcam, 1:500).

The following day, membranes were washed three times in Tris‐Buffered Saline with Tween‐20 (TBST) for 5 min each and then incubated with HRP‐conjugated goat anti‐rabbit secondary antibody (ab6721, Abcam, 1:2000) at room temperature for 1.5 h. After washing in TBST, protein expression levels were detected using a chemiluminescent substrate (NCI4106, Pierce), with GAPDH as the loading control. Band intensity was analyzed using the ImageJ software (Bio‐Rad). Each experiment was repeated three times.

### 2.20 RT‐qPCR

Total RNA was extracted using Trizol reagent (Invitrogen). RNA integrity was confirmed by agarose gel electrophoresis, which displayed clear, sharp 28S and 18S bands, with the 28S band intensity at least twice that of the 18S band, indicating intact RNA. The A260/A280 ratio was 1.8–2.1, suggesting high RNA purity. Reverse transcription was performed using the PrimeScript™ reverse transcription Reagent Kit (Catalog No. RR037A, TaKaRa). RNA precipitate was dissolved in 40 μL RNase‐free water. For each 200 μL RNase‐free tube, 12 μL RNase‐free water, 2 μL oligo(dT), and 3 μL RNA sample were added, mixed, and heated at 70°C for 5 min, followed by rapid cooling in an ice‐water bath for 2 min. Next, 1 μL dNTP, 1 μL guanidine isothiocyanate, 5 μL 5× reverse transcription buffer, and 1 μL Moloney murine leukemia virus reverse transcriptase were added and gently mixed. The reaction was incubated in a 37°C water bath for 90 min, then terminated by heating at 70°C for 5 min, with samples stored on ice.

Target and internal control genes were amplified using a real‐time PCR instrument (ABI 7500, Applied Biosystems). The PCR reaction system consisted of a 25 μL volume, including 2.5 μL 10× PCR buffer, 1.5 μL 25 mmol/L MgCl_2_, 0.5 μL 10 mmol/L dNTP, 0.25 μL 10 mmol/L primers, 1 μL 1 nmol/L probe, 0.25 μL Taq polymerase, and 2.5 μL cDNA, with RNase‐free water added to reach 25 μL. The reaction conditions were as follows: initial denaturation at 94°C for 5 min, followed by 40 cycles of denaturation at 94°C for 30 s, annealing at 58°C for 45 s, and extension at 72°C for 30 s, with a final extension at 72°C for 10 min. Each reaction was performed in triplicate. GAPDH was used as the internal control, and the relative mRNA expression levels of Naa10p, TNF‐α, IL‐6, PGC‐1α, and UCP1 were calculated using the 2^−ΔΔ*Ct*^ method, where ΔΔ*Ct* = Δ*Ct* (experimental group) − Δ*Ct* (control group) and Δ*Ct* = *Ct* (target gene) − *Ct* (internal control). The experiment was repeated three times. Primer sequences are listed in Supporting Information S1: Table S2.

### 2.21 Statistical analysis

Statistical analysis was performed using SPSS version 21.0 (IBM SPSS Statistics). Quantitative data were presented as mean ± SD. For comparisons between two independent groups with normal distribution and homogeneity of variance, an unpaired *t*‐test was used. For comparisons among multiple groups, one‐way Analysis of Variance (ANOVA) followed by Tukey's post‐hoc test was applied. Repeated measures ANOVA with Bonferroni correction was used to compare data among groups at different time points. A *p*‐value of <0.05 was considered statistically significant.

## 3 RESULTS

### 3.1 Identification of key factors in AP using WGCNA and machine learning

To investigate the mechanisms underlying pancreatitis, we established an AP mouse model. Compared to the Con group, AP mice exhibited significantly elevated serum α‐amylase levels (Supporting Information S1: Figure S1A). H&E staining revealed increased inflammatory infiltration and enhanced pathological severity in the pancreatic tissues of AP mice (Supporting Information S1: Figure S1B). Islet cells in the AP group exhibited lipid vacuolization, acinar cells showed loosening and edema, and multinucleated giant cells with “owl's eye” morphology were observed. Additionally, fibrosis was observed around the vasculature and islets, along with amyloid degeneration of acinar cells (Supporting Information S1: Figure S1C). Additionally, RT‐qPCR and western blot analyses showed that levels of the inflammatory factors IL‐6 and TNF‐α were markedly higher in the pancreatic tissues of AP mice compared to the Con group (Supporting Information S1: Figure S1D,E). Biochemical analysis confirmed increased levels of total protein, lipase, ALT, LDH, urinary creatinine, and serum creatinine in AP mice (Supporting Information S1: Figure S1F). These results confirm the successful establishment of the AP mouse model.

**FIGURE 1:**
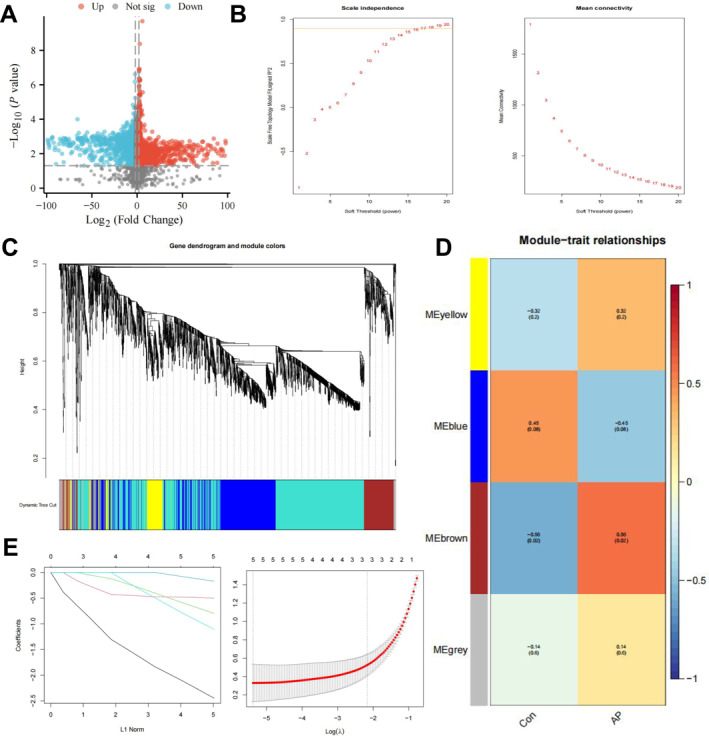
Identification of key factors affecting pancreatitis using weighted gene coexpression network analysis combined with machine learning. (A) Volcano plot of differentially expressed genes in pancreatic tissues from control and pancreatitis mice. (B) Scale independence, average connectivity, and scale‐free topology, with weighted value *β* = 15 selected to satisfy the scale‐free network law. (C) Cluster dendrogram of coexpression network modules. (D) Correlation between clustered gene modules and pancreatitis. (E) LASSO coefficient distribution of differentially expressed genes (left) and selection of the optimal lambda parameter in the LASSO model (right). Sample size in each group, *n* = 10. LASSO, least absolute shrinkage and selection operator.

High‐throughput sequencing was used to investigate changes in gene expression in pancreatic tissues. After normalization, differential expression analysis was performed using the limma package in R, identifying 1637 significantly upregulated genes and 1336 significantly downregulated genes (Figure 1A). To further identify genes closely related to the progression of pancreatitis, we conducted a WGCNA based on the differentially expressed mRNA data. The soft threshold (*β*) was calculated using R, with the optimal *β* value of 15 best fitting a scale‐free network (Figure 1B). With this threshold, we set the minimum module size to 100 and the module merging threshold to 0.5, allowing for dynamic tree cutting (Figure 1C). This process identified four gene modules: MEyellow, MEblue, MEbrown, and MEgrey. The MEgrey module generally represents genes that do not fit into any specific module. Using these four modules, we analyzed the correlation between gene modules and the clinical trait of pancreatitis. The results showed that the MEbrown module had the strongest correlation with pancreatitis, with a coefficient of 0.56 and a *p*‐value less than 0.05 (Figure 1D).

We performed GO and KEGG enrichment analyses on genes in the MEbrown module to better understand the fundamental biological pathways involved in progressing pancreatitis. The results showed significant enrichment of these genes in pathways related to actin organization, cell junctions, and adhesion (Supporting Information S1: Figure S2A). This finding suggests that the development of pancreatitis may involve extensive alterations in cellular structure and signaling networks, particularly in tight junction and adherens junction pathways, which may be closely related to the inflammatory response and cell migration processes in pancreatitis.^27^

To further identify vital regulatory factors associated with pancreatitis, we applied a regression analysis model to fit MEbrown module genes to filter out genes with similar characteristics. Lasso regression was performed using the glmnet function to model the data as a binary classification problem, with sample categories extracted as response variables via regular expression. Model evaluation was conducted by plotting the model object and using cv.glmnet for cross‐validation to determine the optimal lambda value (Figure 1E). Five key factors were identified: Wdr1, Naa10p, Ptpn12, Upf1, and Ralb, all significantly upregulated in pancreatitis mice. Naa10p showed the highest logFC value at 3.10 (Supporting Information S1: Figure S2B). Naa10p encodes N‐α‐acetyltransferase 10, an enzyme in the N‐terminal acetyltransferase family that plays a crucial role in adding acetyl groups to the N‐terminus of newly synthesized proteins, a post‐translational modification essential for protein stability, activity, and subcellular localization.^28^ Our findings suggest that Naa10p plays a key role in the progression of pancreatitis. Among the five characteristic genes identified (Wdr1, Naa10p, Ptpn12, Upf1, and Ralb), only Naa10p exhibited a high logFC value and well‐defined biological function related to acetylation. To date, Wdr1, Ptpn12, and Ralb have no established roles in the PGC‐1α/UCP1 axis. Upf1 may influence PGC‐1α expression or function indirectly via nonsense‐mediated mRNA decay. Therefore, we selected Naa10p for further functional validation.

### 3.2 Elevated Naa10p expression and decreased UCP1 levels in pancreatic tissues of AP mice

**FIGURE 2:**
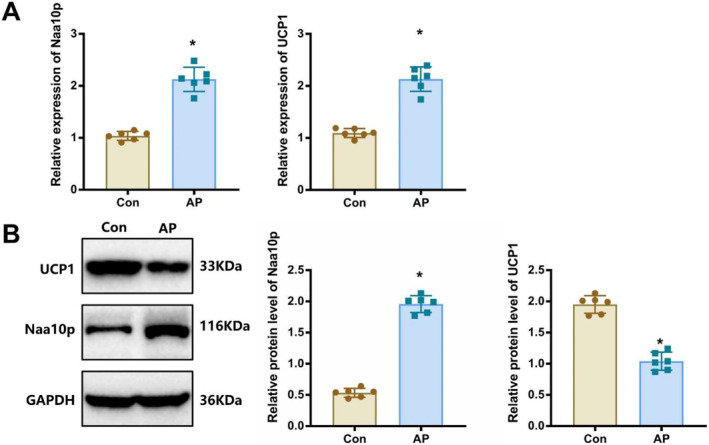
Expression of Naa10p and UCP1 in pancreatic tissues of AP mice. (A and B) RT‐qPCR and western blot analysis of Naa10p and UCP1 levels in pancreatic tissues of each group. * indicates *p* < 0.05 compared to the Con group. The sample size for each group is *n* = 6.

Bioinformatics analysis identified Naa10p as a key gene in the progression of pancreatitis. To confirm this, we examined Naa10p expression in the pancreatic tissues of AP mice. Results showed a significant increase in Naa10p expression in the AP group compared to the Con group (Figure 2A,B). Previous studies have demonstrated that Naa10p can acetylate the N‐terminus of PGC‐1α, thereby preventing its interaction with Pparγ, which, in turn, suppresses the expression of UCP1, a gene essential for beige adipocyte function.^18^ Consistent with this mechanism, we observed a marked decrease in UCP1 expression in the pancreatic tissues of AP mice compared to the Con group (Figure 2A,B). These findings suggest that the high expression of Naa10p in AP pancreatic tissues may contribute to the reduced levels of UCP1.

### 3.3 Silencing Naa10p activates UCP1 expression and alleviates symptoms in AP mice

**FIGURE 3:**
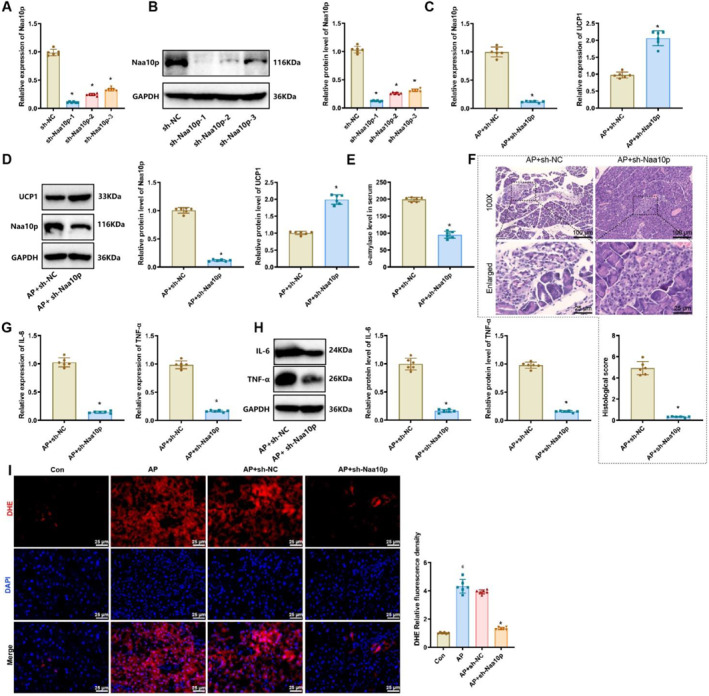
Effects of Naa10p silencing on symptoms in AP mice. (A and B) RT‐qPCR and western blot analysis of Naa10p levels in 266‐6 cells of each group; * indicates *p* < 0.05 compared to the sh‐NC group, with experiments independently repeated three times. (C and D) RT‐qPCR and western blot analysis of Naa10p and UCP1 levels in pancreatic tissues of each group. (E) ELISA analysis of serum α‐amylase levels in each group. (F) H&E staining of pancreatic tissues, scale bar = 100/50 μm. (G and H) RT‐qPCR and western blot analysis of IL‐6 and TNF‐α levels in pancreatic tissues of each group. (I) Dihydroethidium staining for reactive oxygen species levels in pancreatic tissues, scale bar = 25 μm. # indicates *p* < 0.05 compared to the Con group. * indicates *p* < 0.05 compared to the AP+sh‐NC group. The sample size for each group is *n* = 6.

To further investigate the impact of Naa10p on AP symptoms in vivo, we first assessed the silencing efficiency of three different shRNAs targeting Naa10p in 266‐6 cells. Results indicated that sh‐Naa10p‐1 was the most effective, so this shRNA was selected for subsequent experiments (Figure 3A,B). We then injected sh‐Naa10p lentivirus into the tail region of the pancreas in mice prior to cerulein‐induced AP model establishment. RT‐qPCR and western blot analyses showed that, compared to the AP+sh‐NC group, the AP+sh‐Naa10p group exhibited significantly reduced Naa10p levels and a marked increase in UCP1 levels in pancreatic tissues (Figure 3C,D). However, compared to the AP+sh‐NC group, the AP+sh‐Naa10p group exhibited a significant reduction in serum α‐amylase levels (Figure 3E). H&E staining showed that the AP+sh‐Naa10p group had markedly reduced inflammatory infiltration and lower pathological severity in pancreatic tissues (Figure 3F).

Additionally, levels of the inflammatory factors IL‐6 and TNF‐α were significantly decreased in the pancreatic tissues of the AP+sh‐Naa10p group (Figure 3G,H). These results indicate that silencing Naa10p alleviates the symptoms of AP in mice. Moreover, we observed that serum ROS levels were significantly elevated in the AP group compared to the Con group, whereas the AP+sh‐Naa10p group displayed a significant reduction in ROS levels compared to the AP+sh‐NC group (Figure 3I). This suggests that silencing Naa10p mitigates oxidative stress in AP mice.

These findings demonstrate that silencing Naa10p activates UCP1 expression and reduces both inflammation and oxidative stress levels in AP mice.

### 3.4 Naa10p regulates UCP1 expression and affects inflammation and oxidative stress levels in an in vitro AP cell model

**FIGURE 4:**
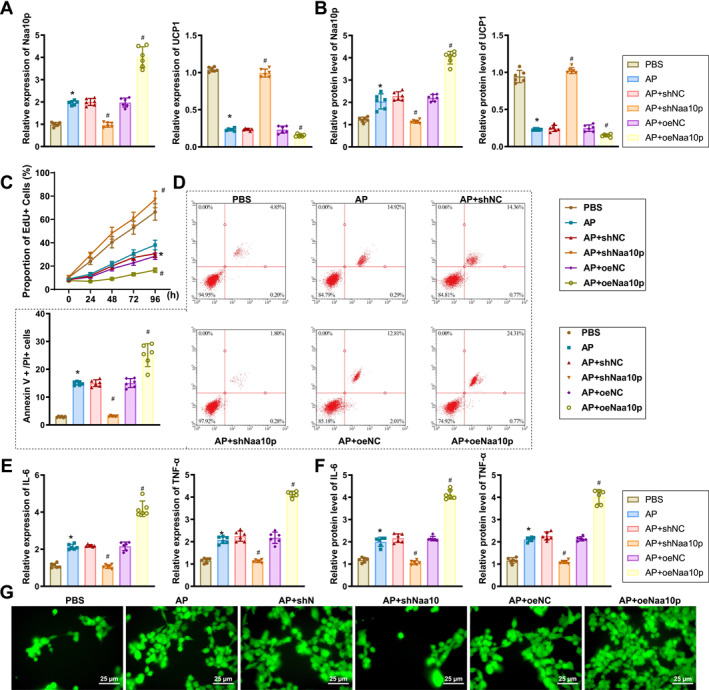
Effects of Naa10p on inflammation and oxidative stress in an in vitro AP cell model. (A and B) RT‐qPCR and western blot analysis of Naa10p and UCP1 expression levels in 266‐6 cells for each group. (C) EdU assay of cell proliferation activity in 266‐6 cells for each group. (D) Flow cytometry analysis of apoptosis and necrosis in 266‐6 cells for each group. (E and F) RT‐qPCR and western blot analysis of IL‐6 and TNF‐α levels in 266‐6 cells for each group. (G) Reactive oxygen species levels in 266‐6 cells for each group, scale bar = 25 μm. * indicates *p* < 0.05 compared to the PBS group. # indicates *p* < 0.05 compared to the AP+sh‐NC or AP+oe‐NC group. Experiments were independently repeated three times. EdU, 5‐Ethynyl‐2′‐deoxyuridine; PBS, phosphate‐buffered saline.

We further investigated the potential mechanism by which Naa10p regulates UCP1 expression in vitro. Using 266‐6 cells, we established an AP cell model and either silenced or overexpressed Naa10p in these cells. RT‐qPCR and western blot analyses revealed that, compared to the PBS group, Naa10p levels were significantly elevated and UCP1 levels significantly decreased in the AP group. Additionally, compared to the AP+sh‐NC group, Naa10p levels were significantly reduced and UCP1 levels significantly increased in the AP+sh‐Naa10p group. Conversely, in the AP+oe‐Naa10p group, Naa10p levels were significantly higher and UCP1 levels were significantly lower than in the AP+oe‐NC group (Figure 4A,B).

EdU assay results indicated that cell proliferation activity in the AP group was significantly reduced compared to the PBS group. In contrast, the AP+sh‐Naa10p group showed a significant increase in cell proliferation compared to the AP+sh‐NC group, whereas the AP+oe‐Naa10p group displayed a significant decrease in cell proliferation compared to the AP+oe‐NC group (Figure 4C).

Flow cytometry analysis revealed that late apoptosis and necrosis (Annexin V+/PI+ cells) were significantly elevated in the AP group compared to the PBS group. However, the AP+sh‐Naa10p group exhibited a marked reduction in late apoptotic and necrotic cells compared to the AP+sh‐NC group, whereas these cells were significantly increased in the AP+oe‐Naa10p group compared to the AP+oe‐NC group (Figure 4D).

Further, RT‐qPCR and western blot analyses showed that levels of inflammatory factors IL‐6 and TNF‐α were significantly higher in the AP group compared to the PBS group. These levels were significantly reduced in the AP+sh‐Naa10p group compared to the AP+sh‐NC group but were elevated in the AP+oe‐Naa10p group compared to the AP+oe‐NC group (Figure 4E,F). Additionally, ROS levels were significantly increased in the AP group compared to the PBS group. The AP+sh‐Naa10p group demonstrated a significant reduction in ROS levels compared to the AP+sh‐NC group, whereas the AP+oe‐Naa10p group showed a marked increase in ROS levels compared to the AP+oe‐NC group (Figure 4G).

These findings indicate that Naa10p regulates UCP1 expression and influences cell proliferation, inflammation, apoptosis, and oxidative stress levels in an in vitro AP cell model.

### 3.5 Naa10p inhibits UCP1 expression by regulating the interaction between PGC‐1α and Pparγ2

**FIGURE 5:**
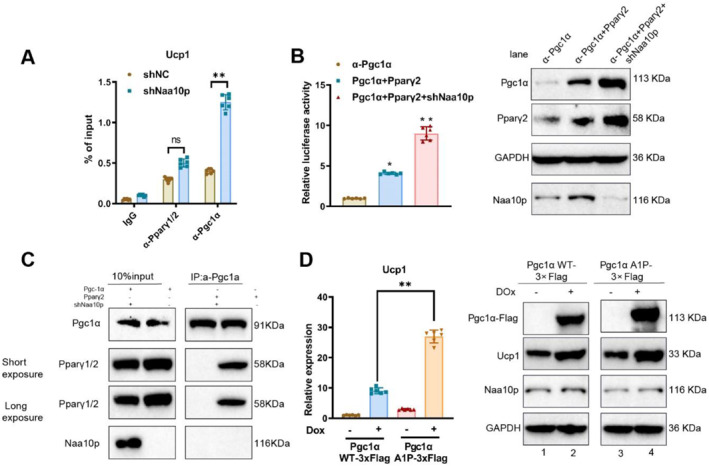
Validation of the mechanism of Naa10p‐regulated UCP1 expression. (A) ChIP‐qPCR analysis of PGC‐1α and Pparγ2 enrichment at the UCP1 promoter in 266‐6 cells, with IgG as a control antibody; NS indicates *p* > 0.05 compared to the sh‐NC group; ** indicates *p* < 0.01 compared to the sh‐NC group. (B) Luciferase reporter assay of UCP1 promoter‐driven luciferase expression in 266‐6 cells, * indicates *p* < 0.05 and ** indicates *p* < 0.01 compared to the PGC‐1α group. (C) Co‐IP analysis of PGC‐1α and Pparγ2 protein interactions in 266‐6 cells for each group. (D) RT‐qPCR and western blot analysis of UCP1 levels in 266‐6 cells, ** indicates *p* < 0.01 compared to the PGC‐1α WT‐3 × Flag group. Experiments were independently repeated three times. Co‐IP, co‐immunoprecipitation.

Using ChIP‐qPCR, we found that silencing Naa10p significantly enhanced the enrichment of PGC‐1α at the UCP1 promoter region, whereas Pparγ2 enrichment at the UCP1 promoter showed only a slight increase (Figure 5A). Consistent with these ChIP results, a luciferase reporter assay driven by the ∼5.5 kb UCP1 promoter demonstrated that silencing Naa10p significantly enhanced the activation of the UCP1 promoter by PGC‐1α and Pparγ2. Moreover, PGC‐1α's activation of the UCP1 promoter required the presence of Pparγ2 (Figure 5B).

Co‐IP experiments showed that silencing Naa10p significantly enhanced the interaction between PGC‐1α and Pparγ2 proteins, indicating that Naa10p inhibits this interaction (Figure 5C). Previous studies have demonstrated that an N‐terminal acetylation mutant of PGC‐1α (PGC‐1α‐A1P), created by substituting the first alanine with proline according to the amino acid rule (X–Pro–X), significantly increases UCP1 mRNA and protein levels.^29^, ^30^ We overexpressed flag‐tagged PGC‐1α wild‐type (PGC‐1α‐WT) and the mutant (PGC‐1α‐A1P) in 266‐6 cells. Results indicated that overexpression of PGC‐1α‐A1P significantly elevated UCP1 mRNA and protein levels (Figure 5D). These findings suggest that Naa10p suppresses UCP1 expression by modulating the interaction between PGC‐1α and Pparγ2.

## 4 DISCUSSION

AP is an acute inflammatory disease with clinical presentations ranging from mild abdominal pain to severe multiorgan dysfunction.^31^ During AP progression, the activation of inflammatory cells and the release of inflammatory mediators are key contributors to pathological damage.^2^, ^9^ Therefore, understanding the molecular mechanisms that control these inflammatory responses, particularly those that regulate cellular metabolism and inflammation, is crucial for developing new therapeutic strategies.^4^, ^32^ This study focuses on the role of Naa10p in AP, specifically its potential mechanism through the PGC‐1α/UCP1 signaling axis.

Naa10p, a protein acetyltransferase, plays roles in various cellular processes, including cell cycle control, metabolic regulation, and inflammatory response.^33^ However, research on Naa10p's role in AP is limited. This study found that Naa10p expression was significantly elevated in the AP model, suggesting that it may play a critical role in AP pathogenesis. Furthermore, genetic silencing of Naa10p resulted in a notable improvement in UCP1 expression levels and reduced inflammatory responses in pancreatic cells, contrasting with previous studies where Naa10p was primarily associated with tumorigenesis and cell proliferation.

The roles of PGC‐1α and UCP1 in maintaining energy balance and regulating mitochondrial function have been widely studied.^34^, ^35^ PGC‐1α is a well‐established transcriptional coactivator that regulates mitochondrial biogenesis,^36^ whereas UCP1 is an uncoupling protein that modulates energy expenditure and thermogenesis. PGC‐1α typically enhances mitochondrial biogenesis, and Pparγ2 may regulate fatty acid oxidation. Naa10p may act as an upstream regulator, integrating intracellular signaling to modulate the activity of this axis. Specifically, Naa10p enhances the stability and transcriptional activity of PGC‐1α via acetylation, which activates nuclear respiratory factors and mitochondrial transcription factor A, promoting mtDNA replication and expression of mitochondrial‐encoded genes, such as components of the respiratory chain. Naa10p may also upregulate fatty acid oxidation‐related genes (e.g., Cpt1b) via Pparγ2, thereby optimizing mitochondrial substrate utilization and increasing adenosine triphosphate production. In this study, we propose that PGC‐1α and Pparγ2 may play novel roles in AP by modulating mitochondrial function to suppress inflammation, extending their functional relevance to nonmetabolic diseases. In future studies, we aim to knock down or overexpress Naa10p/PGC‐1α/Pparγ2 in L‐arginine‐ or caerulein‐induced AP mouse models and assess mitochondrial function (e.g., oxygen consumption rate, mtDNA copy number), inflammatory markers (e.g., IL‐6, myeloperoxidase), and histopathological damage.

The sirtuin family of NAD^+^‐dependent deacetylases plays pivotal roles in metabolic regulation and disease pathogenesis.^37^ Within the PGC‐1α/UCP1 signaling axis, SIRT1 and SIRT3 enhance PGC‐1α function via deacetylation, promoting its binding to nuclear receptors such as PPARγ and ERRα. This activation leads to mitochondrial biogenesis, fatty acid oxidation, and UCP1 expression, thereby regulating thermogenesis and energy metabolism. Dysregulation of this axis is associated with metabolic disorders, including obesity and insulin resistance. In the context of AP, sirtuins exhibit multiple protective effects: SIRT1 reduces inflammatory cytokine release by inhibiting NF‐κB signaling and suppresses macrophage activation via PPARγ activation; SIRT3 improves mitochondrial antioxidant capacity, mitigates oxidative stress, and regulates autophagy and apoptosis in acinar cells. Animal studies have shown that SIRT1 activators (e.g., resveratrol) can alleviate pancreatic damage in AP models, and clinical studies reveal an inverse correlation between serum SIRT1 levels and disease severity.^37^, ^38^ However, the tissue specificity and NAD^+^ dependency of sirtuins present challenges for precise therapeutic targeting, and the roles of other isoforms (e.g., SIRT2, SIRT5, SIRT6) in AP remain unclear. Future studies integrating multiomics technologies are warranted to unravel the underlying molecular networks and develop more selective intervention strategies.

Methodologically, this study combined both in vivo and in vitro experiments to ensure the reliability and broad applicability of the results. In vivo experiments simulated the natural disease environment, whereas in vitro experiments allowed for precise investigation of specific molecular mechanisms under controlled conditions. Additionally, by employing high‐throughput sequencing and advanced bioinformatics methods, we comprehensively analyzed Naa10p‐regulated gene networks, providing novel insights into the disease mechanisms through an integrated methodological approach.

Although this study offers new insights into the role of Naa10p in regulating inflammation in AP, further investigation is needed to elucidate its specific mechanisms and potential interactions compared to other inflammatory regulators, such as NF‐κB or STAT3. For instance, it remains to be determined whether Naa10p directly affects the activity of these factors or influences inflammatory pathways through intermediary molecules.

Our findings suggest that Naa10p modulates the AP PGC‐1α/UCP1 signaling axis, presenting a potential target for new therapeutic strategies. Because of its high incidence and mortality rates, AP poses significant clinical challenges, with current treatments largely limited to supportive and symptomatic care, lacking effective targeted therapies. Naa10p may reduce inflammation and oxidative stress by altering cellular metabolic states, thereby mitigating pancreatic tissue damage. If this molecular mechanism is validated in preclinical and clinical trials, Naa10p and its downstream signaling could become essential therapeutic targets for alleviating AP symptoms, reducing complication risks, and improving patient outcomes. Moreover, further investigation into Naa10p′s regulatory mechanisms may reveal its role in other metabolism‐related inflammatory diseases, providing novel therapeutic avenues for a broad range of inflammatory conditions.

Although this study provides preliminary evidence on the role of Naa10p in AP, it has certain limitations. First, this study primarily relies on animal models, and the findings may only partially translate to humans. Differences in physiology and pathology between mice and humans could impact the clinical applicability of these results. Second, genetic silencing techniques were used in this study, which may introduce nonspecific effects, potentially affecting other unknown signaling pathways. Additionally, this study needed to fully capture the diversity and complexity of clinical pancreatitis cases, such as varying etiologies and disease severity. Therefore, further validation is needed under broader conditions and in larger clinical settings.

Future research should focus on verifying the function and mechanism of Naa10p in AP, mainly through more diverse animal models and human cell applications. Expanding sample sizes and incorporating clinically relevant experimental designs will enhance the generalizability and translational potential of these findings. Moreover, exploring small molecule inhibitors or antagonists targeting Naa10p could provide novel therapeutic options for AP. Understanding the interactions between Naa10p and other inflammatory regulators will also help clarify its position within the inflammatory network, providing a theoretical basis for combination therapies. Ultimately, systematic mechanistic studies and preclinical trials are expected to advance Naa10p‐related therapeutic strategies toward clinical application, offering more effective treatment options for patients with pancreatitis and potentially other inflammatory diseases.

## 5 CONCLUSION

Through constructing an AP mouse model and performing bioinformatics analysis, we identified Naa10p as a key gene in the progression of pancreatitis. Further investigation in both AP mouse and cell models revealed that Naa10p is highly expressed and involved in the progression of pancreatitis, likely by inhibiting UCP1 expression through modulating the interaction between PGC‐1α and Pparγ2, thereby contributing to the disease development (see graphical abstract).

## AUTHOR CONTRIBUTIONS

Jie Du and Zhong Ji conceived and designed this study. Hai Jiang, Taizhe Zhang, and Chuanming Zheng performed the experiments. Taizhe Zhang and Hai Jiang analyzed the data. Jie Du and Hai Jiang wrote the manuscript. All authors reviewed and approved the final version of the manuscript.

## CONFLICT OF INTEREST STATEMENT

The authors declare no conflicts of interest.

## ETHICS STATEMENT

All animal experiments were approved by the Animal Ethics Committee of the First Affiliated Hospital of Bengbu Medical University (Approval No. [2021]238).

## Supporting information

Supporting Information S1

## Data Availability

All data can be provided as needed.
