# Supplementary material for: Naa10p impairs PGC‐1α/Pparγ2 interaction to inhibit mitochondrial protection in pancreatitis
Source: J Cell Commun Signal. 2025 Jun 23;19(2):e70015. doi: 10.1002/ccs3.70015 (PMC12185783; doi:10.1002/ccs3.70015)
Supplement: Supplementary file 1 — Supporting Information S1 [file CCS3-19-e70015-s001.docx]

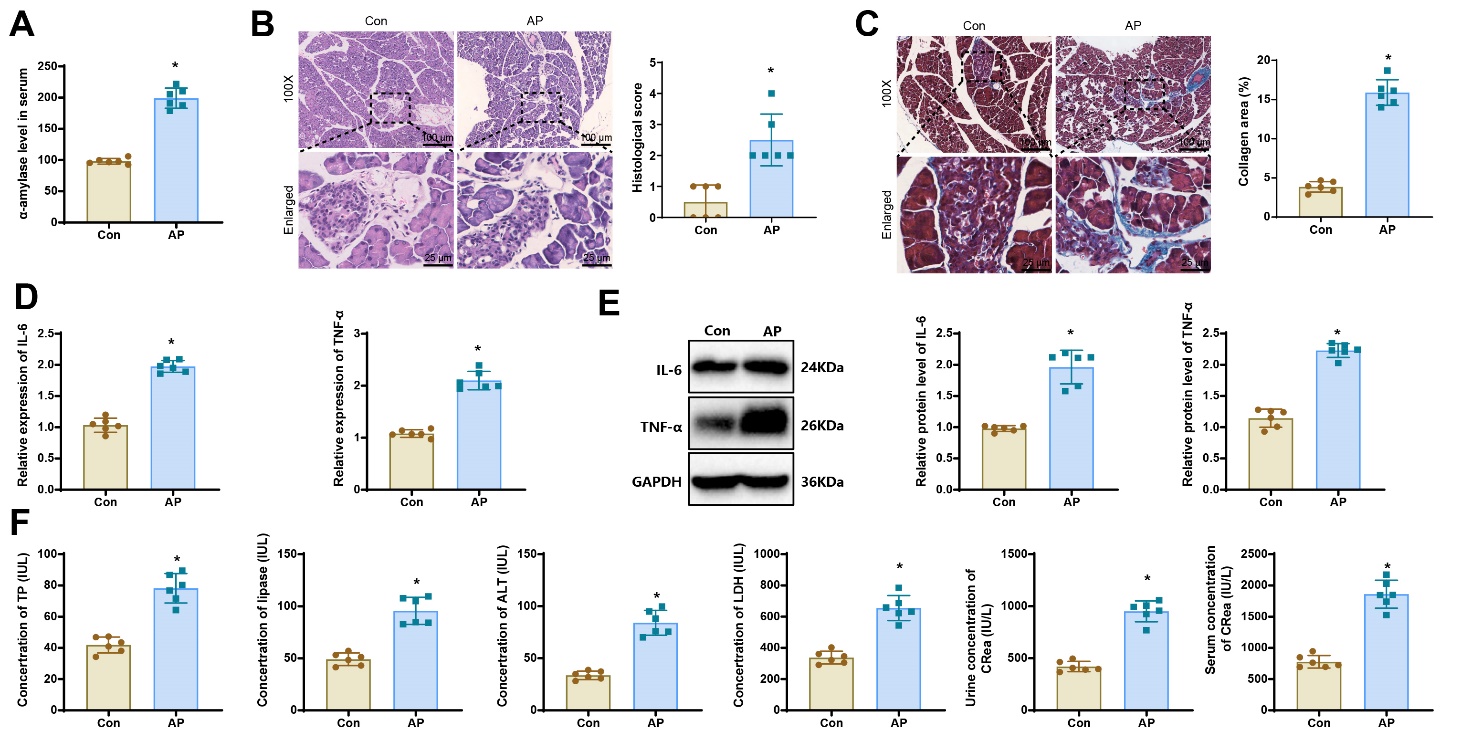


**Figure S1. Validation of AP Mouse Model.**

Note: (A) ELISA measurement of serum α-amylase levels in each group; (B) H&E staining of pancreatic tissues in each group (scale bar = 100 / 25 μm); (C) Masson staining evaluated pancreatic fibrosis (scale bar = 100 / 25 μm); (D-E) Levels of IL-6 and TNF-α in pancreatic tissues measured by RT-qPCR and Western blot in each group; (F) biochemical kits were used to quantify total protein (TP), lipase, ALT, LDH, urinary creatinine, and serum creatinine levels. * indicates *p <* 0.05 compared to the Con group. The sample size for each group is n = 6.


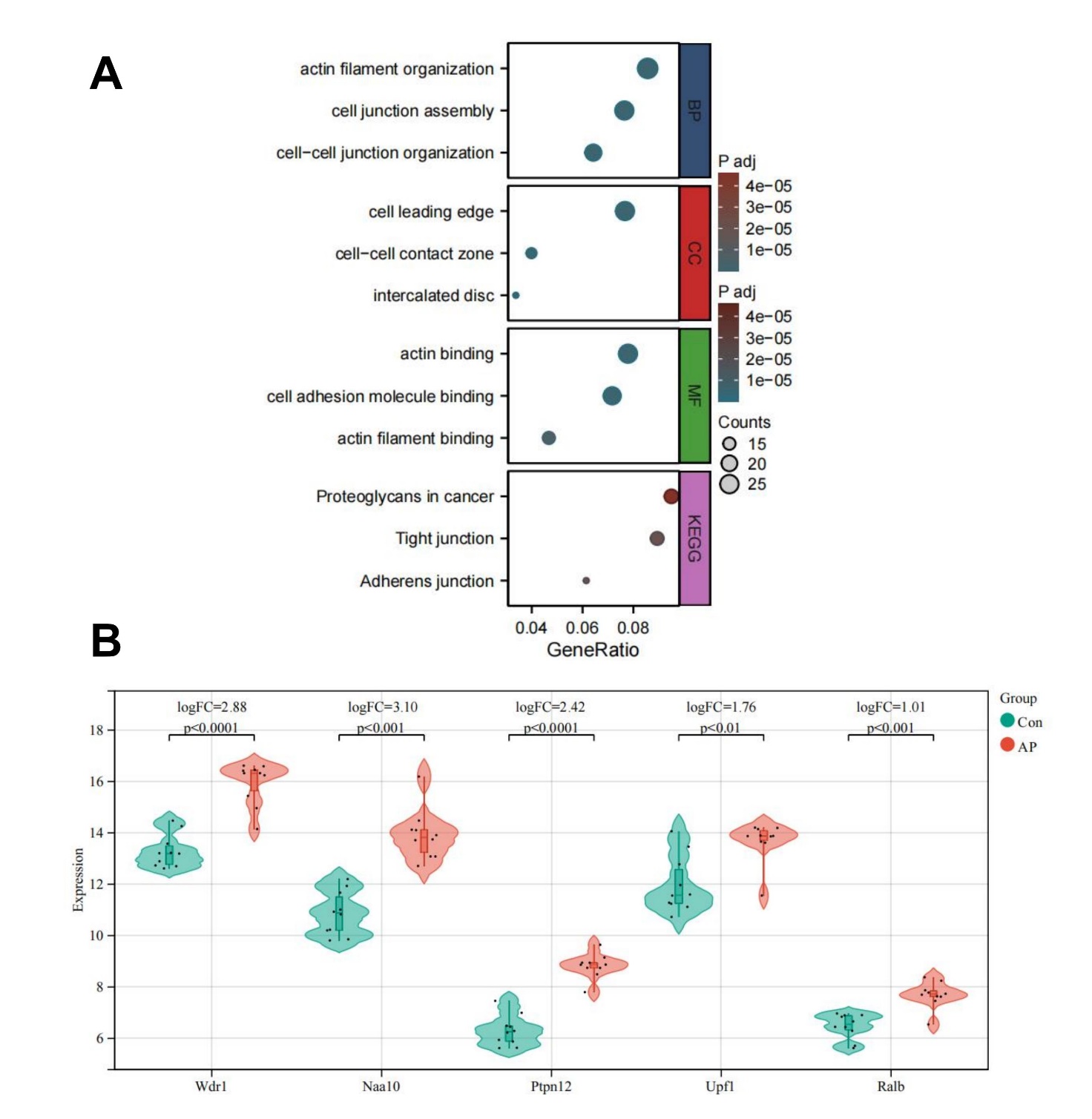


**Figure S2. GO and KEGG Analysis of Pancreatitis-Associated Module Genes and Expression of Characteristic Factors.**

Note: (A) GO and KEGG enrichment analysis results for MEbrown module genes; (B) Expression levels of characteristic factors identified by LASSO analysis. Sample size in each group, n = 10.

**Table S1. Silent sequence.**

| **shRNA** | **Target sequence** |
| --- | --- |
| sh-Naa10p-1 | 5'-CCCAGTGGCCTTGGGAAATTT-3' |
| sh-Naa10p-2 | 5'-ACTACCAGATGAAGTACTATT-3' |
| sh-Naa10p-3 | 5'- AGAGAACTTCAATGCCAAATA-3' |
| sh-NC | 5'-TTCTCCGAACGTGTCACGTA-3' |

**Table S2. PCR Primer Sequence.**

| **Gene** | **Sequence** |
| --- | --- |
| Naa10p | Forward: 5′-ATGAACATCCGCAATG-3′ |
|  | Reverse: 5′-ACAATCTTCCCATTCTC-3′ |
| IL-6 | Forward: 5′-TAACAGATAAGCTGGAGTC-3′ |
|  | Reverse: 5′-TAGGTTTGCCGAGTAGA- 3′ |
| TNF-α | Forward: 5′- GGTGCCTATGTCTCAGCCTCTT -3′ |
|  | Reverse: 5′- GCCATAGAACTGATGAGAGGGAG -3′ |
| PGC-1α | Forward: 5′- GAATCAAGCCACTACAGACACCG -3′ |
|  | Reverse: 5′- CATCCCTCTTGAGCCTTTCGTG -3′ |
| UCP1 | Forward: 5′- GCTTTGCCTCACTCAGGATTGG-3′ |
|  | Reverse: 5′- CCAATGAACACTGCCACACCTC-3′ |
| GAPDH | Forward: 5'- CATCACTGCCACCCAGAAGACTG -3' |
|  | Reverse: 5'- ATGCCAGTGAGCTTCCCGTTCAG -3' |
